# Supplementary material for: SPINK1-induced tumor plasticity provides a therapeutic window for chemotherapy in hepatocellular carcinoma
Source: Nat Commun. 2023 Nov 29;14:7863. doi: 10.1038/s41467-023-43670-9 (PMC10687140; doi:10.1038/s41467-023-43670-9)
Supplement: Supplementary file 1 — Supplementary Information [file 41467_2023_43670_MOESM1_ESM.docx]

**Supplementary Information**

**SPINK1-induced tumor plasticity provides a therapeutic window for chemotherapy in hepatocellular carcinoma**

**Table of content:**

**Supplementary Figure S1-S9**

**Supplementary Table S1. List of target sequences for stable knockdown and knockout of genes used in this study.**

**Supplementary Table S2. List of primers used for qPCR.**

Supplementary References

**
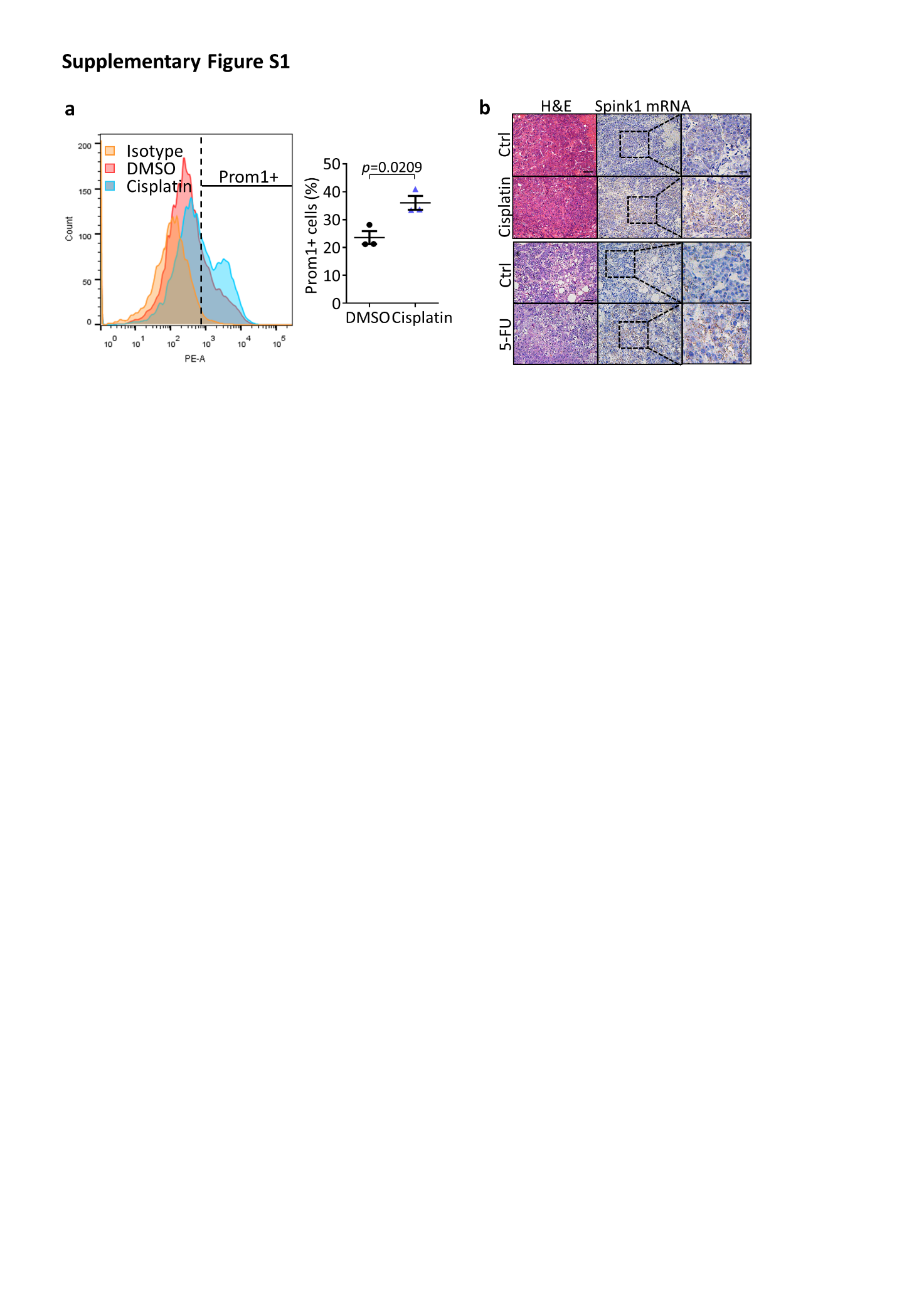
**

**Supplementary Figure S1. (a)** Flow cytometry analysis for Prom1 expression in mouse NRasV12+Myr-AKT HTVI HCC tumors with DMSO or cisplatin treatment. Isotype-stained cells were used as negative control. **(b)** Representative image of livers from NRasV12+Myr-AKT HTVI HCC mice treated with DMSO, 5-FU or cisplatin with Spink1 mRNA staining by RNAScope. Scale bar in low magnification: 50μm. Scale bar in high magnification: 20μm. **(a)** *n* = 3 mice. Data were expressed as mean ± s.e.m. Significance was calculated by **(a)** two-sided Unpaired Student’s *t*-test. Source data are provided as a Source Data file.


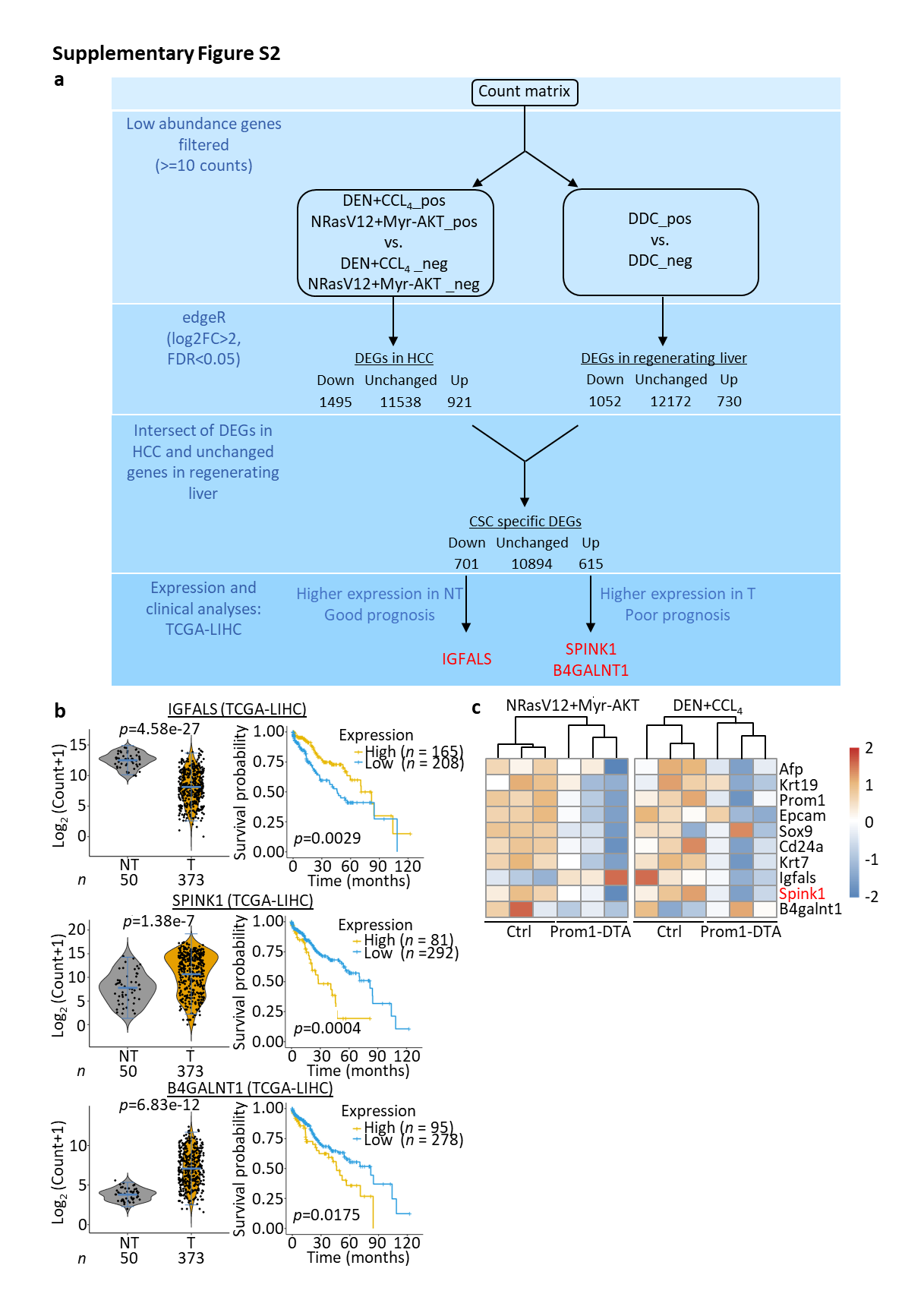


**Supplementary Figure S2. Preferential upregulation of Spink1 in CD133/Prom1+ tumor initiating/propagating cells and HCC tumors in mouse models. (a)** Analytical pipelines for candidates’ selection and criteria in identifying SPINK1 as a tumor initiating/propagating cell specific target in HCC. pos = CD133/Prom1+ cells, neg = CD133/Prom1- cells. **(b)** (Left) Expression of candidate genes in non-tumor (NT) and tumor (T) tissues of TCGA-LIHC cohort. (Right) Kaplan-Meier analysis of the overall survival in HCC patients that segregated by the expression level of candidate genes. **(c)** Heatmap showing expression of selected genes in control (Ctrl) and Prom1-DTA mice of DEN+CCl_4_ and NRasV12+Myr-AKT HCC mouse models, based on transcriptomic sequencing data. *n* = 3 mice. Data was expressed as the range of values from the minimum to the maximum, with the centre represented by the median. The overall shape of the distribution is depicted in the plot. Significance was calculated by **(b)** (left) two-sided Unpaired Student’s *t*-test or (right) log-rank test. Source data are provided as a Source Data file.

**
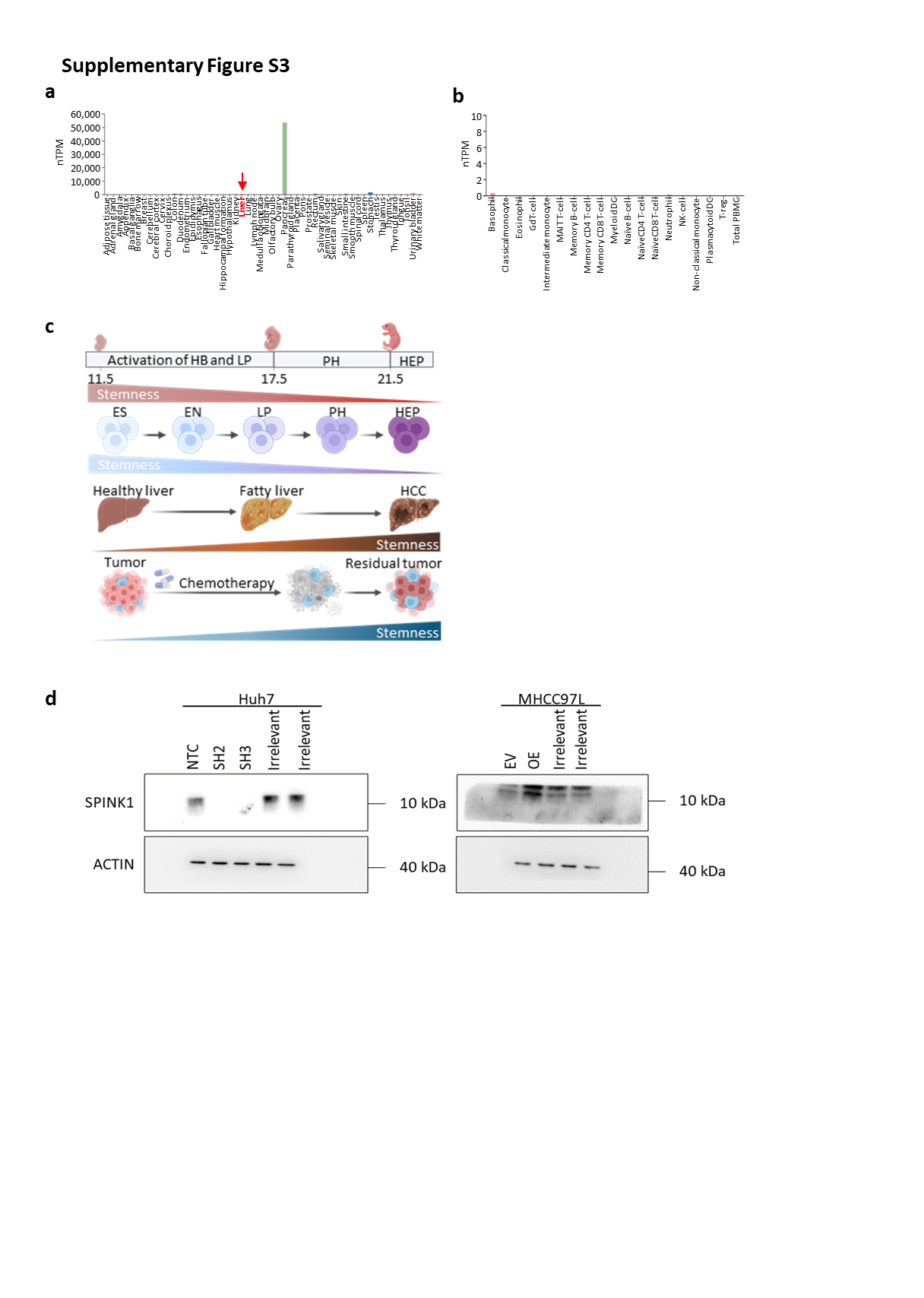
**

**Supplementary Figure S3. (a-b)** *SPINK1* expression in **(a)** various human normal organs and **(b)** immune cells (1). **(c)** Transcriptional profiling of fetal mouse liver from hepatoblasts into adult hepatocytes, of human embryonic stem cells (hESCs) induced to differentiate along hepatic lineages into adult hepatocytes, of normal to HCC livers and of residual HCC tumors from mice treated with 5-FU. Developmental stages: embryonic stem cell (ES), endoderm (EN), liver progenitor cell (LP), and premature hepatocytes (PH), and mature hepatocytes (HEP). **(d)** Validation of SPINK1 expression in Huh7 and MHCC97L cells with or without SPINK1 manipulation by Western blot. Source data are provided as a Source Data file. Illustration for **(c)** was created using BioRender.com.

**
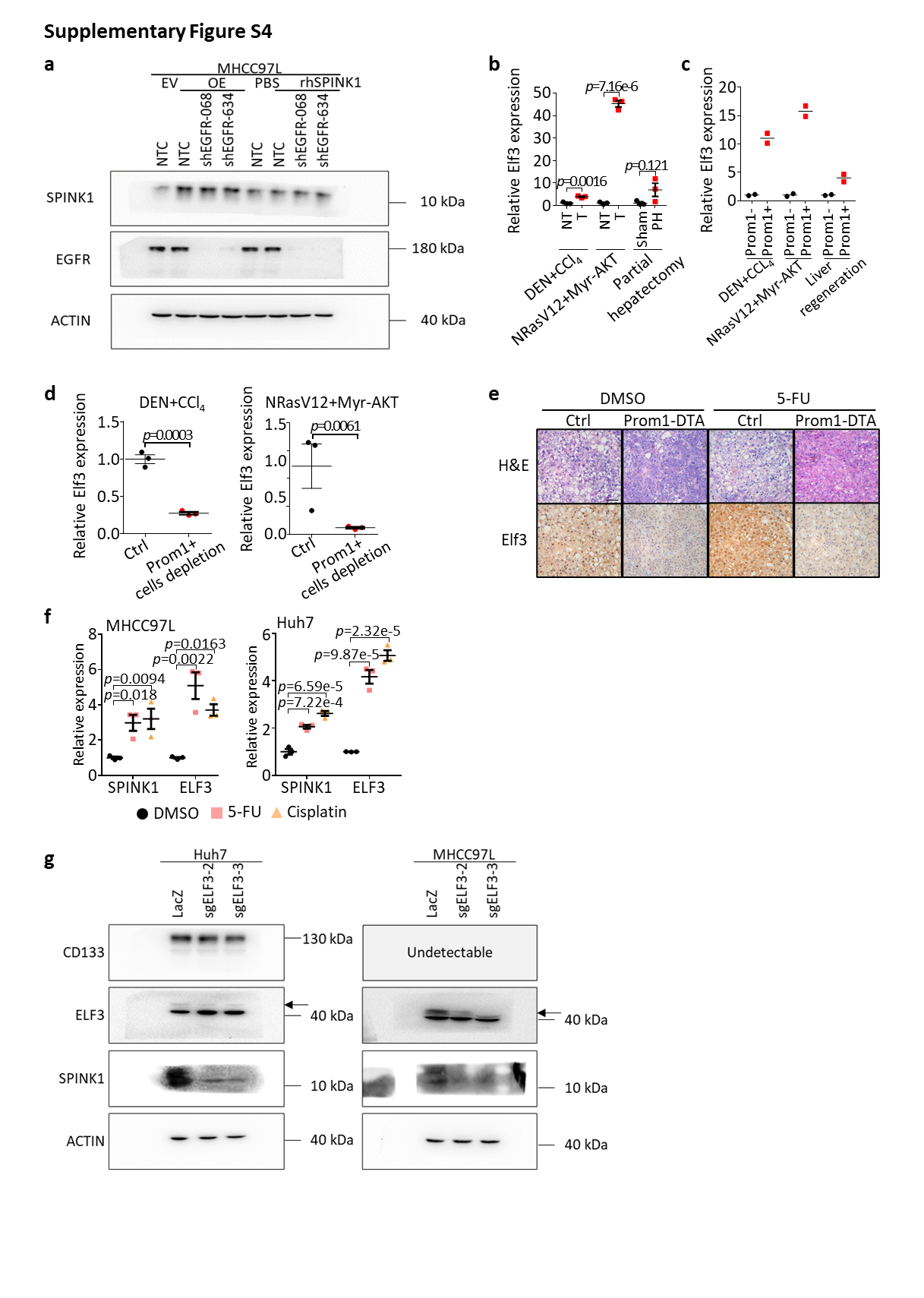
**

**Supplementary Figure S4. (a)** Western Blot validation of SPINK1 and EGFR expressions in MHCC97L cells with or without gene manipulation. **(b)** qPCR analysis for *Elf3* mRNA expression in non-tumor (NT) and tumor (T) tissues of DEN+CCl_4_ and NRasV12+Myr-AKT HCC models, as well as in sham or regenerating livers of partial hepatectomy (PH) mouse model. **(c)** qPCR analysis for expression of *Elf3* in the Prom1+ and Prom1- subpopulations in the two HCC mouse models (DEN+CCl_4_ and NRasV12+Myr-AKT) and one liver regeneration model (0.1% DDC diet). **(d)** qPCR analysis for *Elf3* mRNA expression in control (Ctrl) and Prom1-DTA mice of DEN+CCl_4_ and NRasV12+Myr-AKT HCC mouse models. **(e)** Representative image of livers from Ctrl or Prom1-DTA mice of NRasV12+Myr-AKT HCC mouse model treated with DMSO or 5-FU with ELF3 staining by IHC. Scale bar: 50μm. **(f)** qPCR analysis for *SPINK1* and *ELF3* expression in MHCC97L and Huh7 cells upon DMSO, 5-FU or cisplatin treatment. **(g)** Western Blot validation of ELF3 expression, CD133 and SPINK1 expression in Huh7 and MHCC97L cells with or without gene manipulation. **(b, d)** *n* = 3 mice; **(c)** *n* = 2 mice; **(f)** *n* = 3 independent experiments. Data were expressed as mean ± s.e.m. Significance was calculated by **(b, f)** two-sided Two-way ANOVA with Sidak’s multiple comparison test, or **(d)** two-sided Unpaired Student’s *t*-test. Source data are provided as a Source Data file.

**
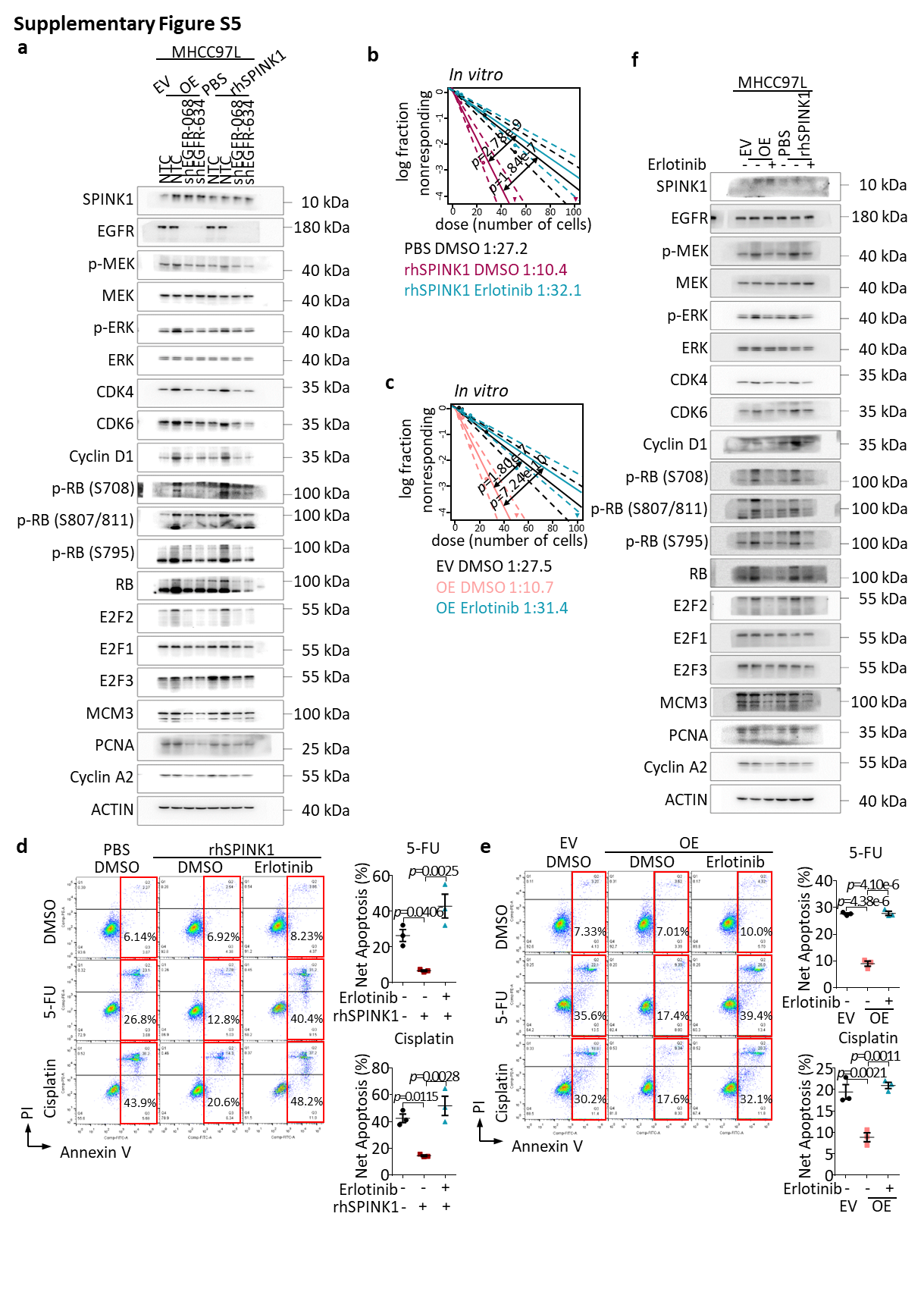
**

**Supplementary Figure S5.** **(a)** Western blot analysis for expression of SPINK1, EGFR, phosphorylated and total MEK, phosphorylated and total ERK, CDK4, CDK6, Cyclin D1, phosphorylated and total Rb, E2F1-3, MCM3, PCNA and Cyclin A2 in MHCC97L cells with EV versus OE versus OE with shRNA against EGFR (shEGFR-068 and shEGFR-634) or PBS versus rhSPINK1 versus shRNA against EGFR (shEGFR-068 and shEGFR-634) with rhSPINK1. **(b-c)** *In vitro* limiting dilution analysis for frequency of TICs of **(b)** MHCC97L with rhSPINK1 treatment or **(c)** SPINK1 overexpression treated with either DMSO or Erlotinib Significance was calculated by Pearson’s χ2 test with 95% confidence intervals. **(d-e)** Cell apoptosis upon 5-FU or cisplatin treatment as demonstrated by Annexin V-PI flow cytometry analysis, in **(d)** MHCC97L cells treated with rhSPINK1 or **(e)** with SPINK1 overexpression with either DMSO or Erlotinib treatment. Significance was calculated by one-way ANOVA. **(f)** Western blot analysis for expression of SPINK1, phosphorylated and total MEK, phosphorylated and total ERK, CDK4, CDK6, Cyclin D1, phosphorylated and total Rb, E2F1-3, MCM3, PCNA and Cyclin A2 in MHCC97L cells with EV with DMSO versus OE with DMSO versus OE with Erlotinib treatment or PBS with DMSO versus rhSPINK1 with DMSO versus Erlotinib treatment with rhSPINK1. **(b-c)** 30 replicates in 3 independent experiments; **(d-e)** *n* = 3 independent experiments. Data were expressed as mean ± s.e.m. Significance was calculated by **(b-c)** one-sided Person’s χ2 test with 95% confidence intervals, or **(d-e)** two-sided Ordinary one-way ANOVA with Tukey’s multiple comparison test. Source data are provided as a Source Data file.

**
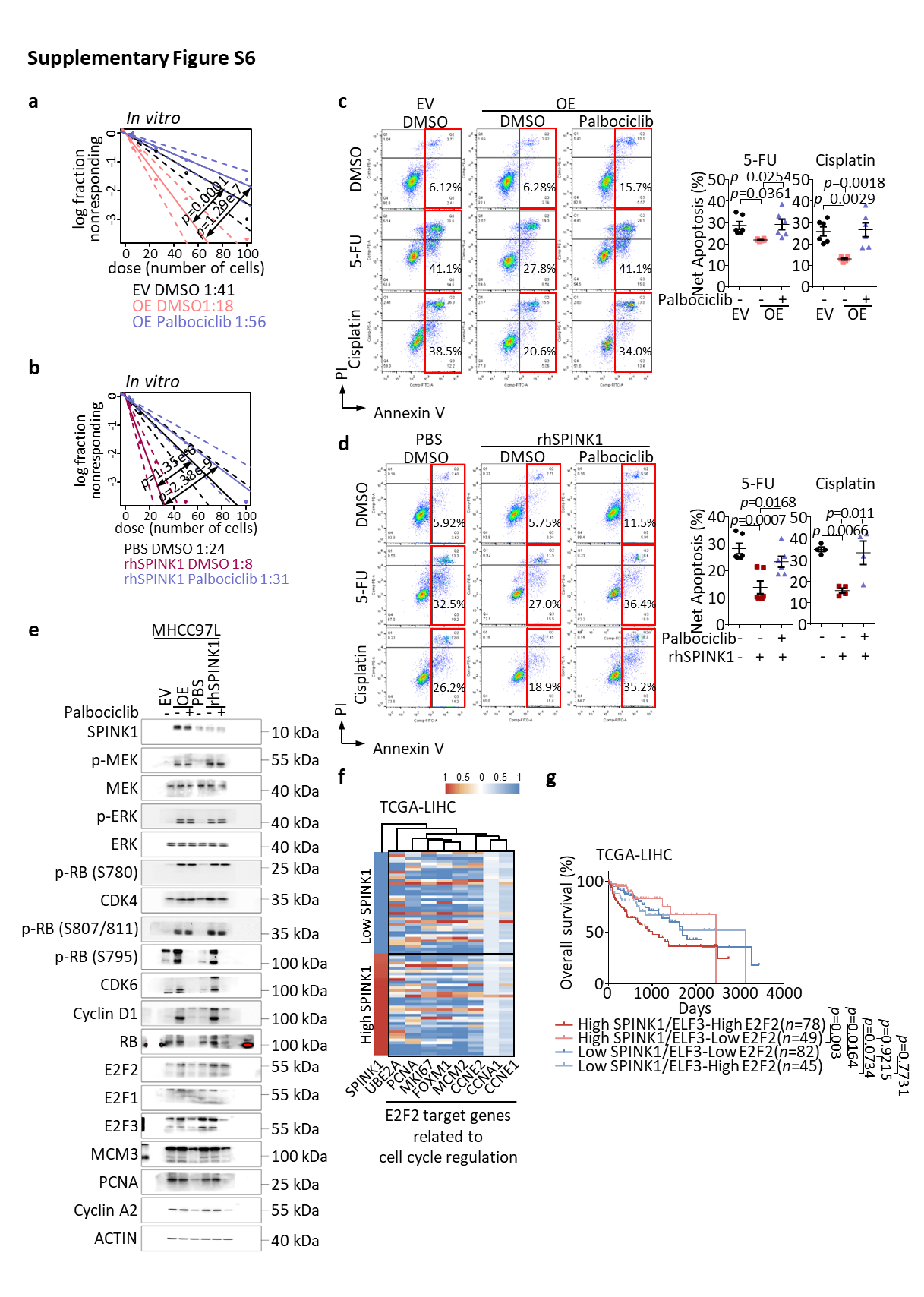
**

**Supplementary Figure S6. (a-b)** *In vitro* limiting dilution analysis for frequency of TICs of **(a)** MHCC97L with rhSPINK1 treatment or **(b)** SPINK1 overexpression treated with either DMSO or Palbociclib. **(c-d)** Cell apoptosis upon 5-FU or cisplatin treatment as demonstrated by Annexin V-PI flow cytometry analysis, in **(c)** MHCC97L cells treated with rhSPINK1 or **(d)** with SPINK1 overexpression with either DMSO or Palbociclib treatment. **(e)** Western blot analysis for expression of SPINK1, phosphorylated and total MEK, phosphorylated and total ERK, CDK4, CDK6, Cyclin D1, phosphorylated and total Rb, E2F1-3, MCM3, PCNA and Cyclin A2 in MHCC97L cells with EV with DMSO versus OE with DMSO versus OE with Palbociclib treatment or PBS with DMSO versus rhSPINK1 with DMSO versus Palbociclib treatment with rhSPINK1. **(f)** Heatmap showing the clustering of tumor *SPINK1* expression level of TCGA-LIHC cohort with E2F2 target genes related to cell cycle regulation. **(g)** Kaplan-Meier curve showing the percentage of overall survival in HCC patients from TCGA-LIHC cohort with high and low *SPINK1*/*ELF3* expression and high and low *E2F2* expression. **(a-b)** 20 replicates in 3 independent experiments; **(c-d)** *n* = 6 independent experiments for 5-FU group in (c-d), 5 independent experiments for cisplatin group in (c) and 4 independent experiments for cisplatin group in (d). Data were expressed as mean ± s.e.m. Significance was calculated by **(a-b)** one-sided Person’s χ2 test with 95% confidence intervals, **(c-d)** two-sided Ordinary one-way ANOVA with Tukey’s multiple comparison test, or **(g)** log-rank test. Source data are provided as a Source Data file.

**
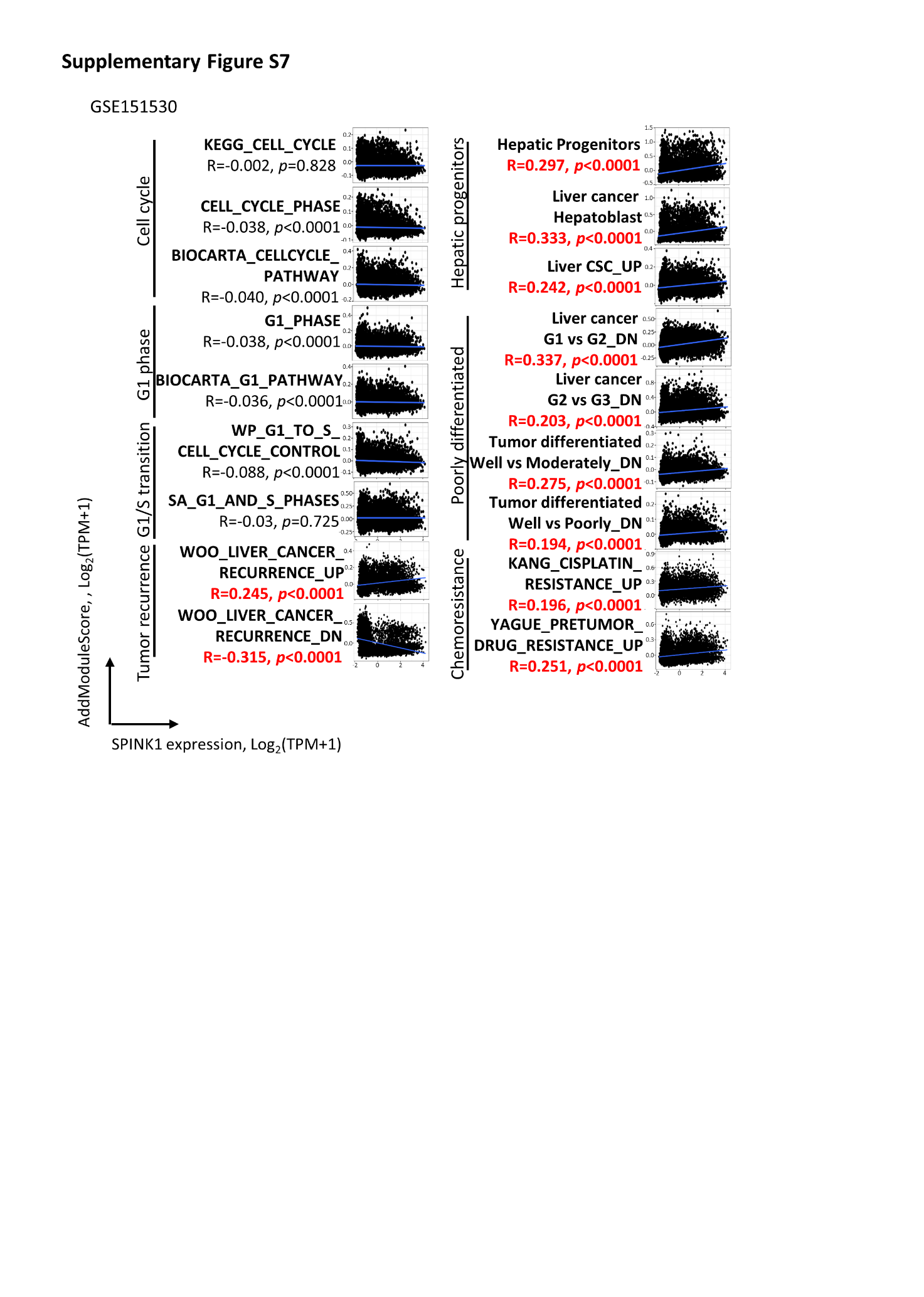
Supplementary Figure S7.** Pearson correlation analyses of SPINK1 expression and scores of gene signatures related to cell cycle, G1 phase, G1/S transition, tumor recurrence, hepatic progenitor, poorly differentiated tumors and chemoresistance from a single-cell atlas of human liver cancer (GSE151530) (2).

**
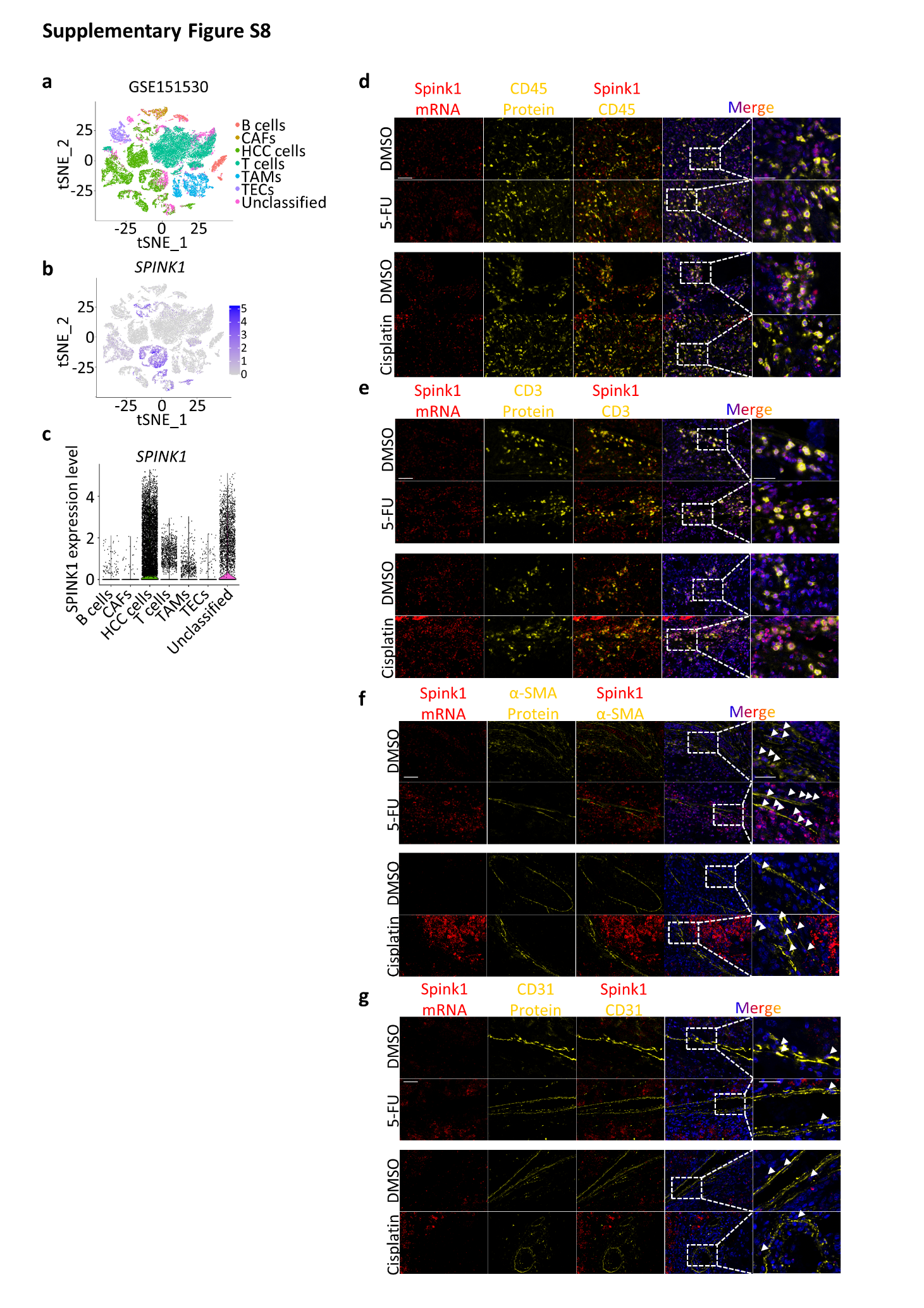
Supplementary Figure S8. (a)** t-SNE plot of single-cells from a single-cell atlas of human liver cancer (GSE151530) (2). Cell types were indicated by colors. Cancer-associated fibroblasts (CAFs), tumor-associated macrophages (TAMs), tumor endothelial cells (TECs). **(b)** t-SNE plot of single-cells in colored by the expression of SPINK1. **(c)** Violin plot of single cells of SPINK1 expression level in different cell types. **(d-g)** Representative image of liver tissues from NRasV12+Myr-AKT HCC mouse model treated with DMSO or 5-FU or cisplatin with Spink1 mRNA (red) and **(d)** CD45 protein (yellow), **(e)** CD3 protein (yellow), **(f)** α-SMA protein (yellow), **(g)** CD31 protein (yellow) staining by RNAScope with co-detection of mRNA and protein. DAPI (blue), nucleus. Cells with co-staining of Spink1 mRNA and α-SMA protein/ CD31 protein indicated by arrowheads. Scale bar in low magnification: 50μm. Scale bar in high magnification: 25μm.

**
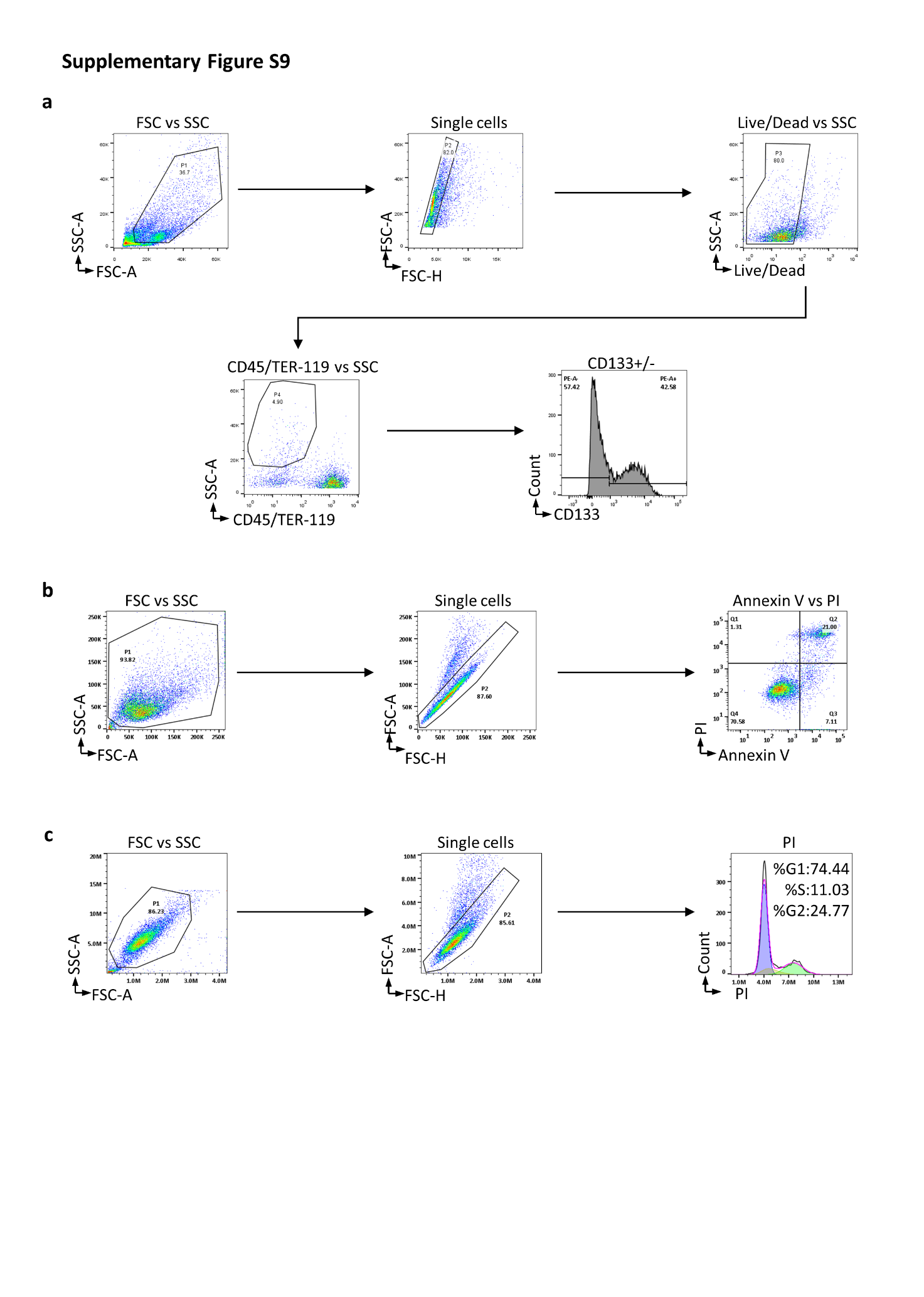
Supplementary Figure S9. (a-c)** Representative gating strategy for **(a)** flow cytometry analysis for Prom1 expression in mouse NRasV12+Myr-AKT HTVI HCC tumors, **(b)** Annexin V-PI flow cytometry analysis and **(c)** cell cycle flow cytometry analysis.

Supplementary Table S1. List of target sequences for stable knockdown and knockout of genes used in this study.

| **Gene Name** | **shRNA (clone)** | **Target sequence** **(5’-3’)*** |
| --- | --- | --- |
| *Knockdown* | | |
| Mouse NTC | shNTC | GATCCGTTCTCCGAACGTGTCACGTTTCAAGAGAACGTGACACGTTCGGAGAACTTTTTTG |
| Mouse Spink1 | shSpink1 | GATCCGCGGGATGTCCCAGAATTTATTTCAAGAGAATAAATTCTGGGACATCCCGCTTTTTTG |
| Human NTC | shNTC | CCGGCAACAAGATGAAGAGCACAACTCGAGTTGGTGCTCTTCATCTTGTTGTTTTT |
| Human SPINK1 | shSPINK1 (clone 2) | CCGGGGCCAAATGTTACAATGAACTCTCGAGAGTTCATTGTAACATTTGGCCTTTTTG |
| Human SPINK1 | shSPINK1 (clone 3) | CCGGGCCAGACTTCTATCCTCATTCCTCGAGGAATGAGGATAGAAGTCTGGCTTTTTG |
| Human EGFR | shEGFR (clone 68) | CCGGGCCACAAAGCAGTGAATTTATCTCGAGATAAATTCACTGCTTTGTGGCTTTTTG |
| Human EGFR | shEGFR (clone 634) | CCGGGCTGGATGATAGACGCAGATACTCGAGTATCTGCGTCTATCATCCAGCTTTTTG |
| *Knockout* | | |
| Human ELF3 | sgELF3 (clone 2) | CACCGCAAGTCATCGGCCCCAAAGGGTTT |
| Human ELF3 | sgELF3 (clone 3) | CACCGCCAGTTCTGGTCGAAGACGCGTTT |

^*^nucleotides complementary to target sequence are underlined

Supplementary Table S2. List of primers used for qPCR.

| Primer Name | Sequence (5’ - 3’) |
| --- | --- |
| Spink1 (mouse)-F | TTTGGCCCTGCTGAGTTTAGC |
| Spink1 (mouse)-R | TGGCATAAGTAATTCCGTCAGTC |
| Elf3 (mouse)-F | GCTGCCACCTGTGAGATCAG |
| Elf3 (mouse)-R | GTGCCAAAGGTAGTCGGAGG |
| Prom1 (mouse)-F | CTCCCATCAGTGGATAGAGAACT |
| Prom1 (mouse)-R | ATACCCCTTTTGACGAGGCT |
| Epcam (mouse)-F | GCGGCTCAGAGAGACTGTG |
| Epcam (mouse)-R | CCAAGCATTTAGACGCCAGTTT |
| Krt19 (mouse)-F | GGGGGTTCAGTACGCATTGG |
| Krt19 (mouse)-R | GAGGACGAGGTCACGAAGC |
| Afp (mouse)-F | AACTTCCTGTCTCAGTCATTCT |
| Afp (mouse)-R | CCTGACATCCAGGTAGATTTCCA |
| Sox9 (mouse)-F | CGGAACAGACTCACATCTCTCC |
| Sox9 (mouse)-R | GCTTGCACGTCGGTTTTGG |
| Bmi (mouse)-F | ATCCCCACTTAATGTGTGTCCT |
| Bmi (mouse)-R | CTTGCTGGTCTCCAAGTAACG |
| Adh1 (mouse)-F | CCATCGAGGACATAGAAGTCGC |
| Adh1 (mouse)-R | TGGTTTCACACAAGTCACCCC |
| G6pc (mouse)-F | CGACTCGCTATCTCCAAGTGA |
| G6pc (mouse)-R | GTTGAACCAGTCTCCGACCA |
| E2f2 (mouse)-F | ACGGCGCAACCTACAAAGAG |
| E2f2 (mouse)-R | GTCTGCGTGTAAAGCGAAGTG |
| Fgfr3 (mouse)-F | GCCTGCGTGCTAGTGTTCT |
| Fgfr3 (mouse)-R | TACCATCCTTAGCCCAGACCG |
| Sphk1 (mouse)-F | GGTGAATGGGCTAATGGAACG |
| Sphk1 (mouse)-R | CTGCTCGTACCCAGCATAGTG |
| Mybl2 (mouse)-F | GGTTTTGAATCCCGACCTTGT |
| Mybl2 (mouse)-R | TGCCATACTTCTTGACCAACTC |
| Actin (mouse)-F | GGCTGTATTCCCCTCCATCG |
| Actin (mouse)-R | CCAGTTGGTAACAATGCCATGT |
| Hprt (mouse)-F | TCAGTCAACGGGGGACATAAA |
| Hprt (mouse)-R | GGGGCTGTACTGCTTAACCAG |
| SPINK1 (human)-F | TCTATCTGGTAACACTGGAGCTG |
| SPINK1 (human)-R | ACACGCATTCATTGGGATAAGT |
| ELF3 (human)-F | GGCCGATGACTTGGTACTGAC |
| ELF3 (human)-R | GCTTGCGTCGTACTTGTTCTTC |
| PROM1 (human)-F | TGGATGCAGAACTTGACAACG |
| PROM1 (human)-R | ATACCTGCTACGACAGTCGTGGT |
| E2F2 (human)-F | CGTCCCTGAGTTCCCAACC |
| E2F2 (human)-R | GCGAAGTGTCATACCGAGTCTT |
| FGFR3 (human)-F | CCCAAATGGGAGCTGTCTCG |
| FGFR3 (human)-R | CCCGGTCCTTGTCAATGCC |
| SPHK1 (human)-F | AGAGTGGGTTCCAAGACACCT |
| SPHK1 (human)-R | GGGTGCAGCAAACATCTCAC |
| MYBL2 (human)-F | CTTGAGCGAGTCCAAAGACTG |
| MYBL2 (human)-R | AGTTGGTCAGAAGACTTCCCT |
| β-actin (human)-F | CATCCACGAAACTACCTTCAACTC |
| β-actin (human)-R | GAGCCGCCGATCCAGACG |

Supplementary References

1. Uhlén, M., Fagerberg, L., Hallström, B. M., Lindskog, C., Oksvold, P., Mardinoglu, A., Sivertsson, Å., Kampf, C., Sjöstedt, E., Asplund, A., Olsson, I., Edlund, K., Lundberg, E., Navani, S., Szigyarto, C. A., Odeberg, J., Djureinovic, D., Takanen, J. O., Hober, S., Alm, T. et al. Tissue-based map of the human proteome. *Science.* **347**, 1260419 (2015).
2. Ma, L., Wang, L., Khatib, S. A., Chang, C. W., Heinrich, S., Dominguez, D. A., Forgues, M., Candia, J., Hernandez, M. O., Kelly, M., Zhao, Y., Tran, B., Hernandez, J. M., Davis, J. L., Kleiner, D. E., Wood, B. J., Greten, T. F., & Wang, X. W. Single-cell atlas of tumor cell evolution in response to therapy in hepatocellular carcinoma and intrahepatic cholangiocarcinoma. *J. Hepatol.* **75**, 1397-1408 (2021).
