# Supplementary material for: SPINK1-induced tumor plasticity provides a therapeutic window for chemotherapy in hepatocellular carcinoma
Source: Nat Commun. 2023 Nov 29;14:7863. doi: 10.1038/s41467-023-43670-9 (PMC10687140; doi:10.1038/s41467-023-43670-9)
Supplement: Supplementary file 3 — Reporting Summary [file 41467_2023_43670_MOESM3_ESM.pdf]

nature portfolio

Corresponding author(s): Prof. Stephanie Ma

Last updated by author(s): 2023-10-31

Reporting Summary

Nature Portfolio wishes to improve the reproducibility of the work that we publish. This form provides structure for consistency and transparency in reporting. For further information on Nature Portfolio policies, see our Editorial Policies and the Editorial Policy Checklist.

Please do not complete any field with "not applicable" or n/a. Refer to the help text for what text to use if an item is not relevant to your study.
For final submission: please carefully check your responses for accuracy; you will not be able to make changes later.

Statistics

For all statistical analyses, confirm that the following items are present in the figure legend, table legend, main text, or Methods section.

n/a	Confirmed	
☐	☒	The exact sample size (*n*) for each experimental group/condition, given as a discrete number and unit of measurement
☐	☒	A statement on whether measurements were taken from distinct samples or whether the same sample was measured repeatedly
☐	☒	The statistical test(s) used AND whether they are one- or two-sided<br>*Only common tests should be described solely by name; describe more complex techniques in the Methods section.*
☒	☐	A description of all covariates tested
☐	☒	A description of any assumptions or corrections, such as tests of normality and adjustment for multiple comparisons
☐	☒	A full description of the statistical parameters including central tendency (e.g. means) or other basic estimates (e.g. regression coefficient) AND variation (e.g. standard deviation) or associated estimates of uncertainty (e.g. confidence intervals)
☐	☒	For null hypothesis testing, the test statistic (e.g. *F*, *t*, *r*) with confidence intervals, effect sizes, degrees of freedom and *P* value noted<br>*Give P values as exact values whenever suitable.*
☒	☐	For Bayesian analysis, information on the choice of priors and Markov chain Monte Carlo settings
☒	☐	For hierarchical and complex designs, identification of the appropriate level for tests and full reporting of outcomes
☐	☒	Estimates of effect sizes (e.g. Cohen's *d*, Pearson's *r*), indicating how they were calculated

Our web collection on statistics for biologists contains articles on many of the points above.

Software and code

Policy information about availability of computer code

Data collection	BD FACSDiva (version 8.0.1) or NovoExpress software (version 1.5.6) was used to collect flow cytometry data.<br>LightCycler480 software (version 1.5.1.62) was used to collect qPCR data.<br>BioRad ImageLab Touch software (version 2.4.0.03) was used to collect Western Blot images.<br>PerkinElmer Vectra PolarisTM Automated Quantitative Pathology Imaging System Phenochart (version 1.0.10) was used to collect immunohistochemistry, Perkin Elmer Victor (version 3) was used for collect the results of ELISA and Luciferase reporter assay.
Data analysis	FlowJo (version 10.7.1) was used for analysis flow cytometry data.<br>LightCycler480 software (version 1.5.1.62) was used for analysis of qPCR data.<br>GraphPad Prism (version 6) was used for analysis data and carry out statistical analysis.<br>GSEA (version 4.3.2) was used for GSEA experiments.<br>ImageJ (version 1.53t), Phenochart (version 1.0.10) and inForm (version 2.4.4) were used to analyze immunohistochemistry.<br>STAR (version 2.5.2), RSEM (version 1.2.31) and EBSeq2 (version 1.10.0) were used for analysis of transcriptome sequencing data.

For manuscripts utilizing custom algorithms or software that are central to the research but not yet described in published literature, software must be made available to editors and reviewers. We strongly encourage code deposition in a community repository (e.g. GitHub). See the Nature Portfolio guidelines for submitting code & software for further information.

Data

Policy information about availability of data

All manuscripts must include a data availability statement. This statement should provide the following information, where applicable:
- Accession codes, unique identifiers, or web links for publicly available datasets
- A description of any restrictions on data availability
- For clinical datasets or third party data, please ensure that the statement adheres to our policy

The TCGA-LIHC publicly available data used in this study are available in the dbGaP repository under accession phs000178.v11.p8 [https://www.ncbi.nlm.nih.gov/projects/gap/cgi-bin/study.cgi?study_id=phs000178.v11.p8].
The transcriptome sequencing data of sorted CD133+ and CD133- cells from DEN+CCl4 HCC model, NRasV12+Myr AKT HCC model and liver regeneration model generated in this study has been deposited in the European Nucleotide Archive (ENA) database under accession code PRJEB59278 [https://www.ebi.ac.uk/ena/browser/view/PRJEB59278]. Reference mouse genome (GRCm38) and splicing junction annotation database are available from University of California Santa Cruz (https://genome.ucsc.edu/cgi-bin/hgGateway?db=mm10), RefSeq (https://www.ncbi.nlm.nih.gov/refseq/), Ensembl (https://grcm38.ensembl.org/index.html).
Previously published Prom1-DTA mouse models transcriptome sequencing data from Zhou et al. is available in the NCBI GEO database under accession code GSE181515 [https://www.ncbi.nlm.nih.gov/geo/query/acc.cgi?acc=GSE181515].
Previously published human liver development scRNA-seq data from Wesley et al. was obtained from http://collections.cellatlas.io/liver-development.
Previously published human HCC scRNA-seq data from Ma et al. have been deposited in NCBI GEO under accession GSE151530 [https://www.ncbi.nlm.nih.gov/geo/query/acc.cgi?acc=GSE151530].
The remaining data are available within the Article, Supplementary Information or Source Data file. Source data are provided with this paper.

Research involving human participants, their data, or biological material

Policy information about studies with human participants or human data. See also policy information about sex, gender (identity/presentation), and sexual orientation and race, ethnicity and racism.

Reporting on sex and gender	For formalin-fixed paraffin-embedded primary human HCC samples, sex and gender were not considered in this study. This study involves 3 males samples.<br><br>For serum samples collected from HCC patients, sex and gender were not considered in this study. This study involves 50 male and 10 female samples.<br><br>For the Tissue microarray (TMA) of HCC patient samples, sex and gender were not considered in this study. This study involves 45 male and 6 female samples.
Reporting on race, ethnicity, or other socially relevant groupings	For formalin-fixed paraffin-embedded primary human HCC samples, race, ethnicity or other socially relevant were not considered in this study and such information is not available.<br><br>For serum samples collected from HCC patients, race, ethnicity or other socially relevant were not considered in this study and such information is not available.<br><br>For the Tissue microarray (TMA) of HCC patient samples, race, ethnicity or other socially relevant were not considered in this study and such information is not available.
Population characteristics	For formalin-fixed paraffin-embedded primary human HCC samples, 3 patients underwent surgery with regular follow-up.<br><br>For serum samples collected from HCC patients, 0 patient underwent surgery.<br><br>For the Tissue microarray (TMA) of HCC patient samples, 51 patients underwent surgery with regular follow-up.
Recruitment	Formalin-fixed paraffin-embedded primary human HCC and serum samples of HCC patients were recruited by the Queen Mary Hospital, Hong Kong. There is no potential bias for recruiting patient cohort.<br><br>HCC patients for tissue microarray (TMA) were recruited by the Sun Yat-sen University Cancer Center, Guangzhou, China), There is no potential bias for recruiting patient cohort.
Ethics oversight	Formalin-fixed paraffin-embedded primary human HCC and adjacent non-tumor liver tissue samples and serum samples of HCC patients were obtained from Queen Mary Hospital with written informed consent obtained from all patients protocol approved by the Institutional Review Board of the University of Hong Kong/ Hospital Authority Hong Kong West Cluster.<br><br>Tissue microarray (TMA) was obtained from Professor Jing-Ping Yun at the Sun Yat-Sen University Cancer Centre in Guangzhou, China, with the approval of the Institutional Review Board for ethical review from the University and consent from patients.

Note that full information on the approval of the study protocol must also be provided in the manuscript.

Field-specific reporting

Please select the one below that is the best fit for your research. If you are not sure, read the appropriate sections before making your selection.

☒ Life sciences ☐ Behavioural & social sciences ☐ Ecological, evolutionary & environmental sciences

Life sciences study design

All studies must disclose on these points even when the disclosure is negative.

Sample size	We did not perform sample size calculations and sample sizes were chosen according to accepted standards in the field of study (Naegle K, Gough NR, Yaffe MB. Criteria for biological reproducibility: what does "n" mean? Sci Signal. 2015 Apr 7;8(371):fs7. doi: 10.1126/scisignal.aab1125.). Individual data points from biological replicates were shown in each figure. For animal studies, preliminary experiments were performed to determine the variation in growth rate and response to treatment. Sample size calculator from the Centre for Comparative Medicine Research (HKU) was used to determine the number of animal with 80% power and p <0.05.
Data exclusions	No data was excluded form this study.
Replication	The number of biological replicates and independent experiments are stated in the figure legend.
Randomization	All mice were randomly assigned to treatment groups to ensure similar initial size of tumor before administration of treatment between group.
Blinding	All authors were not blinded to group allocation, data collection or data analysis because the investigators were responsible for performing the experiment, collecting and labeling the samples, and analyzing the data. For analysis of sequencing data, blinding was not necessary. We have planned the analysis pipeline before the acquisition of sequencing data.

Behavioural & social sciences study design

All studies must disclose on these points even when the disclosure is negative.

Study description	*Briefly describe the study type including whether data are quantitative, qualitative, or mixed-methods (e.g. qualitative cross-sectional,*
Research sample	*State the research sample (e.g. Harvard university undergraduates, villagers in rural India) and provide relevant demographic*
Sampling strategy	*Describe the sampling procedure (e.g. random, snowball, stratified, convenience). Describe the statistical methods that were used to*
Data collection	*Provide details about the data collection procedure, including the instruments or devices used to record the data (e.g. pen and paper,*
Timing	*Indicate the start and stop dates of data collection. If there is a gap between collection periods, state the dates for each sample*
Data exclusions	*If no data were excluded from the analyses, state so OR if data were excluded, provide the exact number of exclusions and the*
Non-participation	*State how many participants dropped out/declined participation and the reason(s) given OR provide response rate OR state that no*
Randomization	*If participants were not allocated into experimental groups, state so OR describe how participants were allocated to groups, and if*

Ecological, evolutionary & environmental sciences study design

All studies must disclose on these points even when the disclosure is negative.

Study description	*Briefly describe the study. For quantitative data include treatment factors and interactions, design structure (e.g. factorial, nested,*
Research sample	*Describe the research sample (e.g. a group of tagged Passer domesticus, all Stenocereus thurberi within Organ Pipe Cactus National*
Sampling strategy	*Note the sampling procedure. Describe the statistical methods that were used to predetermine sample size OR if no sample-size*
Data collection	*Describe the data collection procedure, including who recorded the data and how.*
Timing and spatial scale	*Indicate the start and stop dates of data collection, noting the frequency and periodicity of sampling and providing a rationale for*
Data exclusions	*If no data were excluded from the analyses, state so OR if data were excluded, describe the exclusions and the rationale behind them,*
Reproducibility	*Describe the measures taken to verify the reproducibility of experimental findings. For each experiment, note whether any attempts to*
Randomization	*Describe how samples/organisms/participants were allocated into groups. If allocation was not random, describe how covariates were*
Blinding	*Describe the extent of blinding used during data acquisition and analysis. If blinding was not possible, describe why OR explain why*

Did the study involve field work? ☐ Yes ☐ No

Field work, collection and transport

Field conditions	*Describe the study conditions for field work, providing relevant parameters (e.g. temperature, rainfall).*
Location	*State the location of the sampling or experiment, providing relevant parameters (e.g. latitude and longitude, elevation, water depth).*
Access & import/export	*Describe the efforts you have made to access habitats and to collect and import/export your samples in a responsible manner and in* [illegible]
Disturbance	*Describe any disturbance caused by the study and how it was minimized.*

Reporting for specific materials, systems and methods

We require information from authors about some types of materials, experimental systems and methods used in many studies. Here, indicate whether each material, system or method listed is relevant to your study. If you are not sure if a list item applies to your research, read the appropriate section before selecting a response.

Materials & experimental systems

n/a	Involved in the study
☐	☒ Antibodies
☐	☒ Eukaryotic cell lines
☐	☐ Palaeontology and archaeology
☐	☒ Animals and other organisms
☐	☐ Clinical data
☐	☐ Dual use research of concern
☐	☐ Plants

Methods

n/a	Involved in the study
	☐ ChIP-seq
	☒ Flow cytometry
	☐ MRI-based neuroimaging

Antibodies

Antibodies used

SPINK1 (clone 4D4), 1:100 (Co-IP), 1:1000 (Western blot), Abnova, H00006690-M01
ELF3, 1:100 (ChIP), 1:500 (IHC), 1:1000 (Wester blot) Novus Biologicals, NBP1-30873
EGFR, 1:1000, Cell Signaling Technology, #2232
EGFR (clone D38B1), 1:100 (Co-IP), Cell Signaling Technology, #4267
p-MEK1/2 (Ser217/221), 1:1000, Cell Signaling Technology, #9121
MEK1/2, 1:1000, Cell Signaling Technology, #9122
p-ERK1/2 (Thr202/Tyr204), 1:1000, Cell Signaling Technology, #9101
ERK1/2, 1:1000, Cell Signaling Technology, #9102
CDK4 (clone D9G3E), 1:1000, Cell Signaling Technology, #12790
CDK6 (clone DCS83), 1:1000, Cell Signaling Technology, #3136
Cyclin D1 (clone 92G2), 1:1000, Cell Signaling Technology, #2978
p-RB (Ser708) (clone D59B7), 1:1000, Cell Signaling Technology, #8180
p-RB (Ser795), 1:1000, Cell Signaling Technology, #9301
p-RB (Ser807/811) (clone D20B12), 1:1000, Cell Signaling Technology, #8516
RB (clone 4H1), 1:2000, Cell Signaling Technology, #9309
E2F1 (clone KH95) , 1:500, Santa Cruz, sc-251
E2F2 (clone TFE-25), 1:500, Santa Cruz, sc-9967
E2F3 (clone PG30), 1:500, Santa Cruz, sc-56665
MCM3 (clone D47B6), 1:1000, Cell Signaling Technology, #4003
PCNA (clone PC10), 1:1000, Abcam, ab29
Cyclin A2 (clone E1D9T), 1:1000, Cell Signaling Technology, #91500
beta-ACTIN (clone AC-74), 1:5000, Sigma-Aldrich, A5316
CD133, 1:100, Abcam, ab19898
CD45, 1:100, Abcam, ab10558
CD3 (clone SP7), 1:100, Abcam, ab16669
alpha-SMA, 1:100, Abcam, ab5694
CD31, 1:100, Abcam, ab28364
SPINK1 neutralizing antibody (clone 839304), R&D Systems, 1μg/mL, MAB74961
APC-conjugated CD45 (clone 30-F11), 1:100, eBioscience, 17-0451-83
APC-conjugated TER-119 (clone TER-119), 1:100, eBioscience, 17-5921-83
PE-conjugated CD133 (clone AC133), 1:100, Miltenyi Biotec, 130-080-801

Validation

All antibodies are commercially available and validated by the manufacture as suitable for the applications. The product size in Western Blot experiments were confirmed by comparing the target to protein size ladder.

SPINK1, Abnova, H00006690-M01 (http://www.abnova.com/products/products_detail.asp?catalog_id=H00006690-M01)
ELF3, Novus Biologicals, NBP1-30873 (https://www.novusbio.com/products/elf3-ese-1-antibody_nbp1-30873)
EGFR, Cell Signaling Technology, #2232 (https://www.cellsignal.com/products/primary-antibodies/egf-receptor-antibody/2232)
EGFR, Cell Signaling Technology, #4267 (for co-IP) (https://www.cellsignal.com/products/primary-antibodies/egf-receptor-d38b1-xp-rabbit-mab/4267)
p-MEK1/2 (Ser217/221), Cell Signaling Technology, #9121 (https://www.cellsignal.com/products/primary-antibodies/phospho-mek1-2-ser217-221-antibody/9121)
MEK1/2, Cell Signaling Technology, #9122 (https://www.cellsignal.com/products/primary-antibodies/mek1-2-antibody/9122)
p-ERK1/2 (Thr202/Tyr204), Cell Signaling Technology, #9101 (https://www.cellsignal.com/products/primary-antibodies/phospho-p44-42-mapk-erk1-2-thr202-tyr204-antibody/9101)
ERK1/2, Cell Signaling Technology, #9102 (https://www.cellsignal.com/products/primary-antibodies/p44-42-mapk-erk1-2-antibody/9102)
CDK4, Cell Signaling Technology, #12790 (https://www.cellsignal.com/products/primary-antibodies/cdk4-d9g3e-rabbit-mab/12790)
CDK6, Cell Signaling Technology, #3136 (https://www.cellsignal.com/products/primary-antibodies/cdk6-dcs83-mouse-mab/3136)
Cyclin D1, Cell Signaling Technology, #2978 (https://www.cellsignal.com/products/primary-antibodies/cyclin-d1-92g2-rabbit-mab/2978)
p-RB (Ser708), Cell Signaling Technology, #8180 (https://www.cellsignal.com/products/primary-antibodies/phospho-rb-ser780-d59b7-rabbit-mab/8180)
p-RB (Ser795), Cell Signaling Technology, #9301 (https://www.cellsignal.com/products/primary-antibodies/phospho-rb-ser795-antibody/9301)
p-RB (Ser807/811), Cell Signaling Technology, #8516 (https://www.cellsignal.com/products/primary-antibodies/phospho-rb-ser807-811-d20b12-xp-rabbit-mab/8516)
RB, Cell Signaling Technology, #9309 (https://www.cellsignal.com/products/primary-antibodies/rb-4h1-mouse-mab/9309)
E2F1, Santa Cruz, sc-251 (https://www.scbt.com/p/e2f-1-antibody-kh95)
E2F2, Santa Cruz, sc-9967 (https://www.scbt.com/p/e2f-2-antibody-tfe-25)
E2F3, Santa Cruz, sc-56665 (https://www.scbt.com/p/e2f-3-antibody-pg30)
MCM3, Cell Signaling Technology, #4003 (https://www.cellsignal.com/products/primary-antibodies/mcm3-d47b6-rabbit-mab/4003)
PCNA, Abcam, ab29 (https://www.abcam.com/products/primary-antibodies/pcna-antibody-pc10-ab29.html)
Cyclin A2, Cell Signaling Technology, #91500 (https://www.cellsignal.com/products/primary-antibodies/cyclin-a2-e1d9t-rabbit-mab/91500)
beta-ACTIN, Sigma-Aldrich, A5316 (https://www.sigmaaldrich.com/US/en/product/sigma/a5316)
CD133, Abcam, ab19898 (https://www.abcam.com/products/primary-antibodies/cd133-antibody-stem-cell-marker-ab19898.html)
CD45, Abcam, ab10558 (https://www.abcam.com/products/primary-antibodies/cd45-antibody-ab10558.html)
CD3, Abcam, ab16669 (https://www.abcam.com/products/primary-antibodies/cd3-antibody-sp7-ab16669.html)
alpha-SMA, Abcam, ab5694 (https://www.abcam.com/products/primary-antibodies/alpha-smooth-muscle-actin-antibody-ab5694.html)
CD31, Abcam, ab28364 (https://www.abcam.com/products/primary-antibodies/cd31-antibody-ab28364.html)
SPINK1 neutralizing antibody, R&D Systems, MAB74961 (https://www.rndsystems.com/products/human-spink1-antibody-839304_mab74961)
APC-conjugated CD45, eBioscience, 17-0451-83 (https://www.thermofisher.com/antibody/product/CD45-Antibody-clone-30-F11-Monoclonal/17-0451-82)
APC-conjugated TER-119, eBioscience, 17-5921-83 (https://www.thermofisher.com/antibody/product/TER-119-Antibody-clone-TER-119-Monoclonal/17-5921-83)
PE-conjugated CD133, Miltenyi Biotec, 130-080-801 (https://www.miltenyibiotec.com/HK-en/products/cd133-1-antibody-anti-human-ac133.html#conjugate=pe:size=100-tests-in-200-%C2%B5l)

Eukaryotic cell lines

Policy information about cell lines and Sex and Gender in Research

Cell line source(s)

297T cells (CRL-3216), 297T/17 cells (CRL-11268) and HCC cell lines Hep3B (CRL-8064), HepG2 (CRL-8065), SNU423 (CRL-2238), SNU398 (CRL-2233), SNU449 (CRL-2234) and PLC/PRF/5 (CRL-8024) were purchased from American Type Culture Collection (ATCC).

HCC cell line Huh1 (JCRB0199) and Huh7 (JCRB0403) were purchased from the Japanese Collection of Research Bioresources (JCRB) Cell Bank.

297FT (R70007) cell line was purchased from Invitrogen.

HCC cell lines MHCC97L and MHCC97H were obtained from the Liver Cancer Institute, Fudan University.

Authentication

The cell lines used in this study were authenticated by STR profiling.

Mycoplasma contamination	All cell lines were negative for mycoplasma contamination and routinely tested fro mycoplasma using PCR assay.
Commonly misidentified lines (See ICLAC register)	No commonly misidentified cell lines were used.

Palaeontology and Archaeology

Specimen provenance	*Provide provenance information for specimens and describe permits that were obtained for the work (including the name of the*
Specimen deposition	*Indicate where the specimens have been deposited to permit free access by other researchers.*
Dating methods	*If new dates are provided, describe how they were obtained (e.g. collection, storage, sample pretreatment and measurement), where*

☐ Tick this box to confirm that the raw and calibrated dates are available in the paper or in Supplementary Information.

Ethics oversight	*Identify the organization(s) that approved or provided guidance on the study protocol, OR state that no ethical approval or guidance*

Note that full information on the approval of the study protocol must also be provided in the manuscript.

Animals and other research organisms

Policy information about studies involving animals; ARRIVE guidelines recommended for reporting animal research, and Sex and Gender in Research

Laboratory animals	Mouse (Mus musculus), C57BL/6, 4 weeks old (Liver regeneration mouse model by DDC diet treatment)<br>Mouse (Mus musculus), BALB/AnN-nu, 4-8 weeks old (Liver regeneration mouse model by partial hepatectomy)<br>Mouse (Mus musculus), B6C3F1, 14 days old (DEN+CCl4 fibrosis-induced HCC mouse model)<br>Mouse (Mus musculus), C57BL/6, 8 weeks old (NASH-HCC mouse model)<br>Mouse (Mus musculus), C57BL/6, 6-8 weeks old (HTVI NRAS+AKT HCC mouse model)<br>Mouse (Mus musculus), Prom1C-L/+; Rosa26tdTomato/+ C57BL/6, 4-week-old (Control mouse for Prom1+ cell depletion experiment)<br>Mouse (Mus musculus), Prom1C-L/+; Rosa26DTA/+, 4-week-old (Prom1-DTA mouse for Prom1+ cell depletion experiment)<br>Mouse (Mus musculus), NOD/SCID (NOD.Cg-Prkdcscid IL2rgtm1wjl/SzJ0), 4-6 weeks old (In vivo limiting-dilution and serial transplantation assays)<br><br>All mice were housed in Association for Assessment and Accreditation of Laboratory Animal Care International (AAALAC)-credited facility in 12 hours light/ dark cycle (07:00-19:00 light, 19:00-07:00 dark), with controlled room temperature (23±2°C) and humidity (30-70%) , in groups according to stocking density as recommended in the Guide.
Wild animals	No wild animals were used in the study.
Reporting on sex	Male Mouse (Mus musculus), C57BL/6, 4 weeks old (Liver regeneration mouse model by DDC diet treatment)<br>Male Mouse (Mus musculus), BALB/AnN-nu, 4-8 weeks old (Liver regeneration mouse model by partial hepatectomy)<br>Male Mouse (Mus musculus), B6C3F1, 14 days old (DEN+CCl4 fibrosis-induced HCC mouse model)<br>Male Mouse (Mus musculus), C57BL/6, 8 weeks old (NASH-HCC mouse model)<br>Male Mouse (Mus musculus), C57BL/6, 6-8 weeks old (HTVI NRAS+AKT HCC mouse model)<br>Male Mouse (Mus musculus), Prom1C-L/+; Rosa26tdTomato/+ C57BL/6, 4-week-old (Control mouse for Prom1+ cell depletion experiment)<br>Male Mouse (Mus musculus), Prom1C-L/+; Rosa26DTA/+, 4-week-old (Prom1-DTA mouse for Prom1+ cell depletion experiment)<br>Male Mouse (Mus musculus), NOD/SCID (NOD.Cg-Prkdcscid IL2rgtm1wjl/SzJ0), 4-6 weeks old (In vivo limiting-dilution and serial transplantation assays)<br><br>Male mice were exclusively utilized in all animal experiments of this study, as males exhibit a significantly higher incidence rate of HCC compared to females clinically.
Field-collected samples	No field collected samples were used in the study.
Ethics oversight	License to conduct experiments on animals was obtained from Department of Health, Hong Kong SAR.<br>Approval to conduct animal works at the University of Hong Kong was obtained from Committee on the Use of Live Animals in Teaching and Research (CULATR), the University of Hong Kong

Note that full information on the approval of the study protocol must also be provided in the manuscript.

Clinical data

Policy information about clinical studies

All manuscripts should comply with the ICMJE guidelines for publication of clinical research and a completed CONSORT checklist must be included with all submissions.

Clinical trial registration	*Provide the trial registration number from ClinicalTrials.gov or an equivalent agency.*
Study protocol	*Note where the full trial protocol can be accessed OR if not available, explain why.*
Data collection	*Describe the settings and locales of data collection, noting the time periods of recruitment and data collection.*
Outcomes	*Describe how you pre-defined primary and secondary outcome measures and how you assessed these measures.*

Dual use research of concern

Policy information about dual use research of concern

Hazards

Could the accidental, deliberate or reckless misuse of agents or technologies generated in the work, or the application of information presented in the manuscript, pose a threat to:

No	Yes	
☐	☐	Public health
☐	☐	National security
☐	☐	Crops and/or livestock
☐	☐	Ecosystems
☐	☐	Any other significant area

Experiments of concern

Does the work involve any of these experiments of concern:

No	Yes	
☐	☐	Demonstrate how to render a vaccine ineffective
☐	☐	Confer resistance to therapeutically useful antibiotics or antiviral agents
☐	☐	Enhance the virulence of a pathogen or render a nonpathogen virulent
☐	☐	Increase transmissibility of a pathogen
☐	☐	Alter the host range of a pathogen
☐	☐	Enable evasion of diagnostic/detection modalities
☐	☐	Enable the weaponization of a biological agent or toxin
☐	☐	Any other potentially harmful combination of experiments and agents

Plants

Seed stocks	*Report on the source of all seed stocks or other plant material used. If applicable, state the seed stock centre and catalogue number. If*
Novel plant genotypes	*Describe the methods by which all novel plant genotypes were produced. This includes those generated by transgenic approaches,*
Authentication	*Describe any authentication procedures for each seed stock used or novel genotype generated. Describe any experiments used to*

ChIP-seq

Data deposition

☐ Confirm that both raw and final processed data have been deposited in a public database such as GEO.

☐ Confirm that you have deposited or provided access to graph files (e.g. BED files) for the called peaks.

Data access links<br>*May remain private before publication.*	*For "Initial submission" or "Revised version" documents, provide reviewer access links. For your "Final submission" document, provide a link to the deposited data.*

Files in database submission | *Provide a list of all files available in the database submission.*

Genome browser session (e.g. UCSC) | *Provide a link to an anonymized genome browser session for "Initial submission" and "Revised version" documents only, to enable peer review. Write "no longer applicable" for "Final submission" documents.*

Methodology

Replicates | *Describe the experimental replicates, specifying number, type and replicate agreement.*

Sequencing depth | *Describe the sequencing depth for each experiment, providing the total number of reads, uniquely mapped reads, length of reads and*

Antibodies | *Describe the antibodies used for the ChIP-seq experiments; as applicable, provide supplier name, catalog number, clone name, and lot number.*

Peak calling parameters | *Specify the command line program and parameters used for read mapping and peak calling, including the ChIP, control and index files*

Data quality | *Describe the methods used to ensure data quality in full detail, including how many peaks are at FDR 5% and above 5-fold enrichment.*

Software | *Describe the software used to collect and analyze the ChIP-seq data. For custom code that has been deposited into a community*

Flow Cytometry

Plots

Confirm that:

☒ The axis labels state the marker and fluorochrome used (e.g. CD4-FITC).

☒ The axis scales are clearly visible. Include numbers along axes only for bottom left plot of group (a 'group' is an analysis of identical markers).

☒ All plots are contour plots with outliers or pseudocolor plots.

☒ A numerical value for number of cells or percentage (with statistics) is provided.

Methodology

Sample preparation | HCC cells were harvested by trysin and stained with specific antibody in FACS buffers.
Tumor cells harvested from HCC mouse models by liver dissociation kit, mouse (Miltenyi Biotec, #130105807) and stained with specific antibody in FACS buffers.

Instrument | BD FACSDiva (version 8.0.1), NovoExpress software (version 1.5.6)

Software | FlowJo (version 10.7.1)

Cell population abundance | The final target population consists of at least 10,000 cells

Gating strategy | The total population was visualized by FSC/SSC graph, and the main population was gated.
Subsequently, singlets were gated using FSH-H/FSC-A graph.
The singlets were then used for analysis.

☒ Tick this box to confirm that a figure exemplifying the gating strategy is provided in the Supplementary Information.

Magnetic resonance imaging

Experimental design

Design type | *Indicate task or resting state; event-related or block design.*

Design specifications | *Specify the number of blocks, trials or experimental units per session and/or subject, and specify the length of each trial*

Behavioral performance measures | *State number and/or type of variables recorded (e.g. correct button press, response time) and what statistics were used*

Acquisition

Imaging type(s) — *Specify: functional, structural, diffusion, perfusion.*

Field strength — *Specify in Tesla*

Sequence & imaging parameters — *Specify the pulse sequence type (gradient echo, spin echo, etc.), imaging type (EPI, spiral, etc.), field of view, matrix size,*

Area of acquisition — *State whether a whole brain scan was used OR define the area of acquisition, describing how the region was determined.*

Diffusion MRI ☐ Used ☐ Not used

Preprocessing

Preprocessing software — *Provide detail on software version and revision number and on specific parameters (model/functions, brain extraction,*

Normalization — *If data were normalized/standardized, describe the approach(es): specify linear or non-linear and define image types used for*

Normalization template — *Describe the template used for normalization/transformation, specifying subject space or group standardized space (e.g.*

Noise and artifact removal — *Describe your procedure(s) for artifact and structured noise removal, specifying motion parameters, tissue signals and*

Volume censoring — *Define your software and/or method and criteria for volume censoring, and state the extent of such censoring.*

Statistical modeling & inference

Model type and settings — *Specify type (mass univariate, multivariate, RSA, predictive, etc.) and describe essential details of the model at the first and*

Effect(s) tested — *Define precise effect in terms of the task or stimulus conditions instead of psychological concepts and indicate whether ANOVA*

Specify type of analysis: ☐ Whole brain ☐ ROI-based ☐ Both

Statistic type for inference — *Specify voxel-wise or cluster-wise and report all relevant parameters for cluster-wise methods.*

(See Eklund et al. 2016)

Correction — *Describe the type of correction and how it is obtained for multiple comparisons (e.g. FWE, FDR, permutation or Monte Carlo).*

Models & analysis

n/a	Involved in the study
☐	☐ Functional and/or effective connectivity
☐	☐ Graph analysis
☐	☐ Multivariate modeling or predictive analysis

Functional and/or effective connectivity — *Report the measures of dependence used and the model details (e.g. Pearson correlation, partial correlation,*

Graph analysis — *Report the dependent variable and connectivity measure, specifying weighted graph or binarized graph,*

Multivariate modeling and predictive analysis — *Specify independent variables, features extraction and dimension reduction, model, training and evaluation*

This checklist template is licensed under a Creative Commons Attribution 4.0 International License, which permits use, sharing, adaptation, distribution and reproduction in any medium or format, as long as you give appropriate credit to the original author(s) and the source, provide a link to the Creative Commons license, and indicate if changes were made. The images or other third party material in this article are included in the article's Creative Commons license, unless indicated otherwise in a credit line to the material. If material is not included in the article's Creative Commons license and your intended use is not permitted by statutory regulation or exceeds the permitted use, you will need to obtain permission directly from the copyright holder. To view a copy of this license, visit http://creativecommons.org/licenses/by/4.0/
